# Single-cell RNA sequencing and spatial transcriptomic analysis reveal a distinct population of G6PD^+^ cells with aberrant bile acid metabolism in hepatocellular carcinoma

**DOI:** 10.3389/fimmu.2026.1739293

**Published:** 2026-02-02

**Authors:** Xing Jiang, Haiyan Quan, Ting Yin, Hailun Yao, Yajun Li, Bin Peng, Xinye Yuan, Weiguang Zeng, Honghui Chen, Rong Li

**Affiliations:** 1 Hunan Provincial Key Laboratory of Basic and Clinical Pharmacological Research of Gastrointestinal Cancer, Department of Pharmacy, Institute of Pharmacy and Pharmacology, the Second Affiliated Hospital, University of South China, Hengyang, Hunan, China; 2 School of Pharmaceutical Technology, Hunan Polytechnic of Environment and Biology, Hengyang, Hunan, China

**Keywords:** bile acids, G6PD, hepatocellular carcinoma, machine learning, tumor microenvironment

## Abstract

**Background:**

Metabolic reprogramming is a hallmark of hepatocellular carcinoma (HCC). Among various metabolic pathways, bile acids act not only as crucial metabolites but also as key signaling molecules that regulate diverse physiological and pathological processes in the liver. However, the biological functions and clinical implications of bile acid metabolism in HCC progression remain largely unclear.

**Methods:**

Single-cell transcriptomic data from 67 patients with HCC were integrated to construct a bile acid metabolism scoring system. Pseudotime trajectory analysis was employed to characterize the differentiation patterns of cells exhibiting abnormal bile acid metabolism. Spatial transcriptomics was used to explore their spatial distribution features. Furthermore, machine learning algorithms were applied to analyze transcriptomic data from HCC cohorts to develop a prognostic prediction model. The findings were complemented by immune infiltration analysis, molecular characterization, and drug sensitivity prediction using CellMiner, followed by molecular docking validation.

**Results:**

G6PD^+^ malignant tumor cells with high bile acid metabolism scores exhibited enhanced bile acid metabolic activity, accompanied by activation of macrophages and endothelial cells. These cells were predominantly localized at the tumor boundary region. A prognostic prediction model based on G6PD^+^ expression successfully identified a high-risk subgroup with significantly poorer outcomes. *In vitro* experiments demonstrated that knockdown or overexpression of G6PD markedly affected the proliferative, migratory, and invasive capacities of HCC cells.

**Conclusion:**

This study reveals that bile acid metabolism promotes HCC progression by facilitating vascular network formation and establishing an immunosuppressive tumor microenvironment. The bile acid metabolism scoring system may serve as a novel prognostic biomarker and provide a theoretical foundation for developing precision therapeutic strategies in HCC.

## 1 Introduction

Hepatocellular carcinoma (HCC) is a highly malignant tumor with substantial global incidence and mortality, typically arising in the setting of liver cirrhosis, chronic hepatitis virus infection, or metabolic dysfunction–associated fatty liver disease (MAFLD) (1). To sustain their rapid proliferation, HCC cells undergo profound metabolic reprogramming, which has become one of the defining hallmarks of their malignant phenotype (2). Among these alterations, a striking feature is their strong dependence on aerobic glycolysis — even under oxygen-rich conditions, tumor cells preferentially obtain energy via glycolysis, a phenomenon known as the Warburg effect. This process leads to excessive lactate production and secretion, thereby creating an acidic tumor microenvironment that promotes immune evasion and tumor growth (3, 4). Compared with normal hepatocytes, HCC cells exhibit markedly increased expression of glycolysis-related genes such as GLUT1, HK2, and PKM2, which enhances glucose uptake and utilization, accelerates tumor progression, and correlates with poor patient outcomes (5, 6). Beyond glycolysis, metabolic abnormalities in HCC also encompass the pentose phosphate pathway, fatty acid metabolism, and amino acid metabolism (2, 7). Consequently, targeting metabolic reprogramming has emerged as a critical strategy in the development of novel HCC therapies.

The liver serves as the central organ for bile acid synthesis and metabolism. In addition to their well-known role in digestion, bile acids act as pivotal signaling molecules that regulate metabolism, inflammation, and immune responses, thereby exerting widespread influence on hepatic physiological and pathological processes (8, 9). Disruption of bile acid homeostasis—whether in synthesis, transport, or secretion—can lead to intrahepatic cholestasis, persistent hepatocellular injury, and chronic inflammation, ultimately predisposing the liver to malignant transformation (10, 11). In addition, cholesterol homeostasis mediated by bile acid metabolism can also promote tumorigenesis (12).

Clinically, this connection is supported by the observation that up to 40% of HCC patients present with cholestatic jaundice at diagnosis, suggesting a strong association between bile acid dysregulation, tumor progression, and adverse prognosis (13). Mechanistically, loss of farnesoid X receptor (FXR) disrupts the feedback regulation of bile acid synthesis, leading to excessive accumulation, chronic hepatic injury, and spontaneous HCC development (14, 15). Conversely, FXR activation restores bile acid homeostasis by repressing the rate-limiting enzyme CYP7A1 and inducing the bile salt export pump BSEP, thereby alleviating hepatic inflammation and injury (16). Collectively, bile acid dysregulation represents a fundamental pathogenic driver of HCC. However, whether there exist distinct cellular subtypes with abnormal bile acid metabolism in HCC tissues, and whether these can serve as independent prognostic indicators, remains to be further investigated.

In this study, we integrated bulk, single-cell, and spatial transcriptomic datasets to develop an innovative bile acid metabolism scoring system and comprehensively characterize bile acid metabolism in HCC at the molecular and spatial levels. We identified a unique subset of G6PD^+^ malignant cells exhibiting aberrant bile acid metabolism, which are closely associated with angiogenesis and the establishment of an immunosuppressive microenvironment. By fulfilling these findings, we seek to establish a mechanistic link between bile acid metabolic reprogramming and HCC progression, providing a translatable framework for targeting this pathway.

## 2 Materials and methods

### 2.1 Data sources

We systematically explored multiple biological dimensions of HCC by integrating data from four complementary public databases. Single-cell transcriptomic datasets were obtained from GSE149614 (n=10 patients) and GSE156625 (n=57 patients), which reveal the cellular heterogeneity of HCC (17, 18). Spatial transcriptomic data (10x Genomics, 226334_D5_2) provided a tissue-level spatial context for molecular expression patterns. In addition, bulk transcriptomic data from TCGA-LIHC, ICGC-LIRI-JP, GSE14520, and GSE43619 cohorts were used to validate the robustness of our findings across diverse clinical populations. The bile acid metabolism-related gene set was curated from the GeneCards database (**Supplementary Table 1**), encompassing comprehensive pathway components to ensure biological relevance. This study involved the analysis of existing, de-identified human genomic and transcriptomic data obtained from public repositories. All data were accessed and used in accordance with the respective database policies. Therefore, no separate ethics approval was required for this secondary analysis, as confirmed by our institutional review board.

### 2.2 Single-cell RNA sequencing analysis

Single-cell RNA sequencing (scRNA-seq) data were processed using the Seurat package (v4.3.0) in the R environment. To ensure high data quality, genes expressed in fewer than three cells were excluded, and cells with fewer than 50 detected genes or with mitochondrial gene content exceeding 5% were filtered out. Data normalization was performed using the SCTransform method, followed by IntegrateData to correct batch effects and construct a unified analytical object. Dimensionality reduction was achieved via principal component analysis (PCA), and cells were clustered using the FindNeighbors and FindClusters functions to identify subpopulations with similar transcriptional profiles. Cluster visualization was accomplished through Uniform Manifold Approximation and Projection (UMAP). Cell types were annotated based on cluster-specific marker genes identified by FindAllMarkers, with each subpopulation defined by its unique transcriptional signature (19). The irGSEA function was applied to score bile acid metabolism activity, enabling evaluation of functional enrichment across distinct cellular subtypes. This comprehensive analytical workflow ensured robust identification and functional characterization of the cellular components within the tumor microenvironment.

### 2.3 Cell–cell communication analysis

To systematically dissect the complex intercellular communication network within the tumor microenvironment, we employed CellChat, an integrative computational framework containing a manually curated ligand–receptor interaction database and downstream signaling pathway annotations (20). This approach enabled the identification and quantification of significantly enriched ligand–receptor interactions based on co-expression patterns across cell types. Through this rigorous framework, we uncovered previously unrecognized signaling axes that coordinate intercellular communication in the HCC microenvironment, providing mechanistic insights into how specific cellular subpopulations cooperate to promote tumor progression and immune evasion.

### 2.4 Single-cell trajectory construction

To capture the dynamic evolution of cellular states during tumor progression, we performed pseudotime trajectory inference on epithelial cells using Monocle (v2.30.1) (21). The analysis focused on epithelial subsets previously integrated into the R environment. A new cell dataset was created using newCellDataSet, and genes dynamically expressed along pseudotime were identified. Dimensionality reduction was achieved using the DDRTree algorithm implemented in reduceDimension, mapping cells onto a minimum spanning tree while preserving developmental relationships inferred from transcriptomic similarity. The resulting trajectory was visualized with plot_cell_trajectory, illustrating gene expression dynamics along pseudotime. Pseudotime-dependent genes were further characterized, and their expression trends were visualized using plot_pseudotime_heatmap, thereby depicting the molecular reprogramming events accompanying epithelial phenotypic transitions in HCC.

### 2.5 Spatial transcriptomics analysis

Spatial transcriptomic data were processed and visualized using the Seurat R package. Data normalization was performed with SCTransform, followed by unsupervised clustering to identify spatially distinct transcriptional domains. Cell-type annotation was validated through histopathological inspection of H&E-stained sections. To achieve accurate cell identity mapping within spatial coordinates, we applied a multimodal computational framework combining Robust Cell Type Decomposition (RCTD) and Multimodal Intersection Analysis (MIA), integrating reference scRNA-seq datasets with spatial transcriptomic profiles (22). This multimodal integration preserved the native spatial context of cell–cell interactions and revealed previously unrecognized region-specific cellular distributions within the HCC microenvironment.

### 2.6 Machine learning algorithms

An ensemble machine learning framework was employed to construct a comprehensive prognostic model for HCC. Ten algorithms were systematically evaluated, including random survival forests, elastic net regression, Lasso regression, stepwise Cox regression, ridge regression, CoxBoost, partial least squares regression for Cox models, supervised principal component analysis, gradient boosting machines, and survival support vector machines (23). The model with the highest average concordance index (C-index) across all comparisons was selected as the final predictive model. Supervised Principal Component Analysis (SuperPC) was selected as the final modeling algorithm based on its superior average Harrell’s Concordance Index (C-index). The model’s key hyperparameters specifically, the correlation cutoff (threshold) for feature gene selection and the number of principal components were optimized via a grid search combined with 10-fold cross-validation on the training set, with the combination maximizing the cross-validated C-index chosen for the final model refit. To ensure generalizability and prevent overfitting, we relied on SuperPC’s inherent dimensionality reduction regularization, conducted all tuning via cross-validation to avoid bias, and most critically, validated the fixed model on an independent testing cohort where it achieved a C-index of 0.71, demonstrating consistent performance. Model performance was rigorously validated using time-dependent receiver operating characteristic (ROC) curves, and the area under the curve (AUC) was calculated via the timeROC package. Multivariate Cox regression analysis confirmed that the risk score derived from this model was an independent and statistically significant prognostic factor for patient outcomes.

### 2.7 Immune infiltration and survival analysis

To comprehensively characterize the immune landscape of HCC, we conducted gene set variation analysis (GSVA) in combination with deconvolution approaches implemented in the IOBR platform (24, 25). Enrichment patterns of various immune cell subsets were systematically assessed, and expression differences in immune checkpoint molecules and functional immune signatures were compared between predefined subgroups. The clinical relevance of the prognostic model was further evaluated by survival analyses across multiple HCC cohorts (GEO, LIHC and ICGC datasets) using the survival (v3.5-8) and Survminer (v0.4.9) packages. Patients were stratified into high- and low-risk groups based on the median risk score, and Kaplan–Meier survival curves were plotted to compare outcomes between groups.

### 2.8 Drug sensitivity prediction

To identify potential therapeutic compounds, we performed integrative drug sensitivity modeling using the CellMiner database. Gene expression matrices were log_2_-transformed and batch-corrected for normalization before integration with drug response data. The resulting computational model predicted candidate compounds with potential therapeutic relevance. Structural modeling and molecular docking were conducted using Discovery Studio 2019. Protein crystal structures were obtained from the Protein Data Bank (PDB, ID: 7UAG), and small-molecule structures from PubChem (ID: 321674-73-1). After structural optimization, molecular docking simulations were carried out with rigorously defined parameters to characterize the binding modes between candidate compounds and their target proteins.

### 2.9 Cell culture and functional assays

The HCC lines HCCLM3 were sourced from the Cell Bank of Shanghai Institutes for Biological Sciences, Chinese Academy of Sciences, with identity validation performed by Short Tandem Repeat (STR) analysis. HCCLM3 cells were grown in DMEM containing 10% FBS and 1% streptomycin/penicillin at 37°C in a 5% CO_2_ incubator. To evaluate clonogenic potential, HCCLM3 cells were plated at 6- or 24-well plates. Following 7 days of culture in 6-well plates, colonies were fixed in 4% paraformaldehyde, stained with crystal violet, and imaged under a microscope. For EdU assay, follow the operating steps of the EdU kit (C0071S; Beyotime; China). To specifically assess cell migration and minimize the contribution of proliferation in scratch assay, cells were cultured in medium containing 0.5% FBS for the duration of the assay. The wound closure was monitored by time-lapse microscopy at the same pre-marked locations at 0-, 12-, and 24-hours post-scratch. Small interfering RNA (siRNA)-mediated knockdown of G6PD was performed to validate its functional role. The target sequence for si-G6PD in our study as follows:

si-G6PD-1: 5’-GCTGAAGAAGTATGACAACATTTCAAGAGAATGTTGTCATA-3’, si-G6PD-2: 5’-GCAGTTCGTGTGGGTGATAAATTCAAGAGATTTATCACCCA-3’, si-G6PD-3: 5’-GGACAAGCCCATCATTCACAGTTCAAGAGACTGTGAATGA-3’. Cells were seeded in appropriate culture plates and grown to 60-70% confluence. Transfection was carried out using Lipofectamine 3000 according to the manufacturer’s instructions, with a final siRNA concentration of 50 nM. After 48 hours of transfection, cells were harvested for subsequent functional assays and Western blot analysis. All *in vitro* experiments were performed with at least three independent biological replicates. Quantitative data are presented as the mean ± standard deviation (SD).

### 2.10 Cell migration and invasion

Cell migration was evaluated using a wound healing assay. Briefly, cells were seeded in 6-well plates and grown to full confluence. A straight scratch was created using a sterile pipette tip, and the detached cells were gently washed away with PBS. Images of the wound were captured at the same location at 0, 12, and 24 hours under a microscope. The wound area at each time point was measured using Image J software, and the changes in area were analyzed to quantify cell migration ability.

Cell invasion was assessed using Transwell chambers (8 μm). The upper chambers were pre-coated with Matrigel matrix, and cells resuspended in serum-free medium were seeded into the upper compartment. The lower chambers were filled with medium containing 10% FBS as a chemoattractant. After 24 hours of incubation, non-invaded cells on the upper surface of the membrane were carefully removed with a cotton swab. The cells that had invaded to the lower side were fixed with 4% paraformaldehyde, stained with 0.1% crystal violet, and then counted under a microscope in randomly selected fields for statistical analysis.

### 2.11 Statistical analysis

All statistical analyses were performed using R software (v4.3.1) and GraphPad Prism (v8.0). Kaplan–Meier survival analysis with log-rank testing was used to compare survival differences between groups. Continuous variables were analyzed using two-tailed Student’s t-tests, and correlations were assessed using Spearman’s rank correlation. Statistical significance was defined as *p* < 0.05.

## 3 Results

### 3.1 Single-cell transcriptomic features of HCC patients

**Figure 1:**
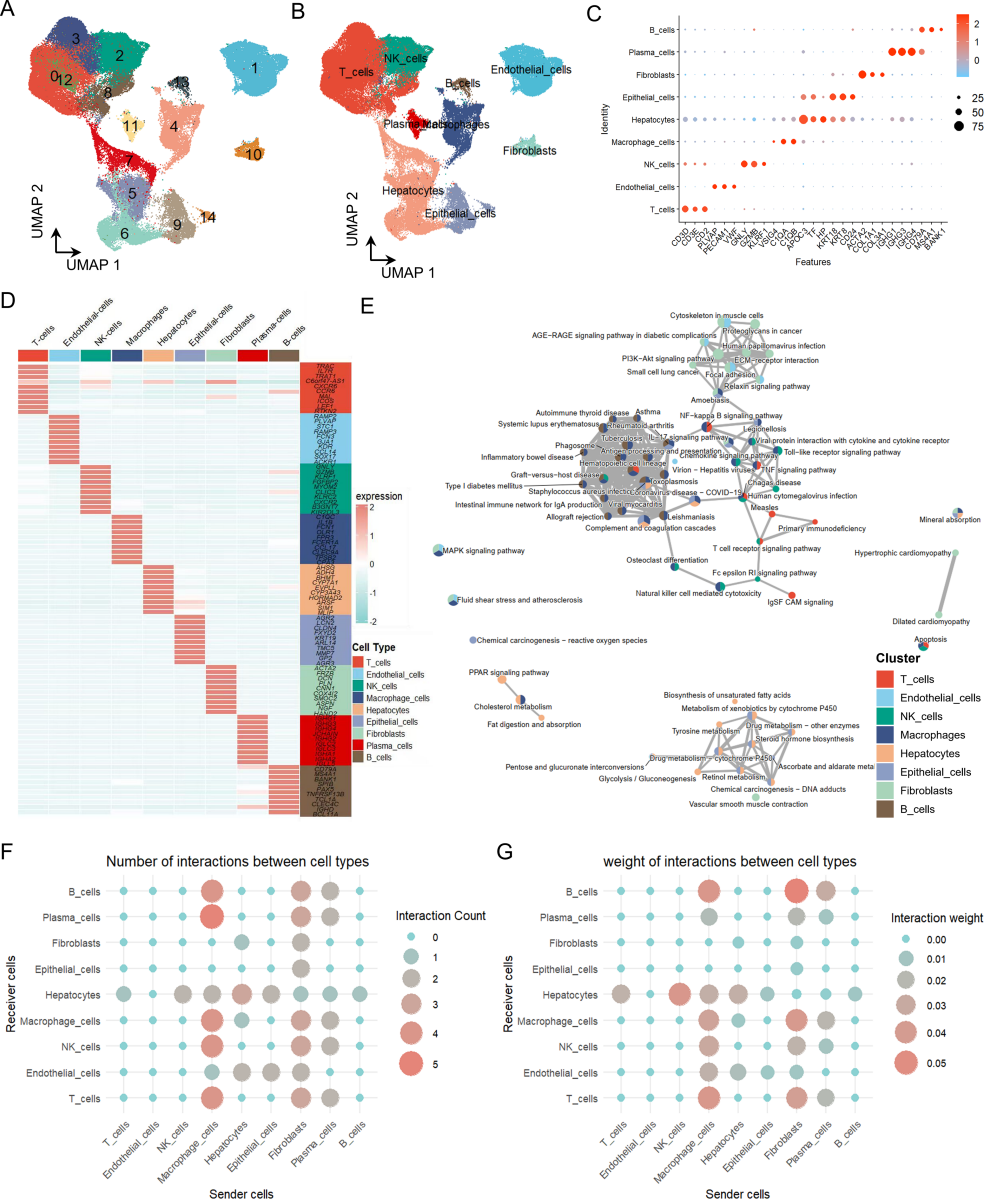
Single-cell RNA sequencing analysis of HCC patients. **(A)** UMAP visualization of cell clusters derived from scRNA-seq data of HCC samples. **(B)** UMAP projection showing annotated cell types within the HCC tumor microenvironment. **(C)** Bubble plot illustrating the expression of marker genes across identified cell populations. **(D)** Heatmap displaying the top 10 characteristic genes for each cell type. **(E)** Network representation of KEGG pathway enrichment analysis across cell types. **(F, G)** CellChat analysis depicting intercellular communication networks within the HCC microenvironment.

To comprehensively characterize the heterogeneity of the HCC tumor microenvironment, we integrated the single-cell RNA sequencing datasets GSE149614 and GSE156625. After standard QC filtering (as described in Methods), we retained a total of 94,900 high-quality cells and 34,326 genes for downstream analysis. Through unsupervised clustering, we identified nine distinct cellular populations with unique transcriptional signatures (**Figures 1A, B**), including T cells, B cells, NK cells, endothelial cells, fibroblasts, macrophages, plasma cells, hepatocytes, and epithelial cells. These clusters exhibited characteristic expression patterns of canonical marker genes (**Figure 1C**), reflecting population-specific transcriptional identities and functional states (**Figure 1D**). Functional enrichment analysis further revealed that these cell populations were significantly enriched in biological processes such as cell adhesion, immune activation, and metabolic pathways (**Figure 1E**). CellChat analysis delineated a complex network of ligand–receptor interactions within the HCC microenvironment, highlighting the extensive intercellular communication among diverse cell types (**Figures 1F, G**). Notably, myeloid-derived cells exhibited particularly strong signaling activity, underscoring their central role as communication hubs that orchestrate cross-talk between immune and stromal components in the tumor microenvironment.

### 3.2 Enhanced bile acid metabolism in malignant HCC cells

**Figure 2:**
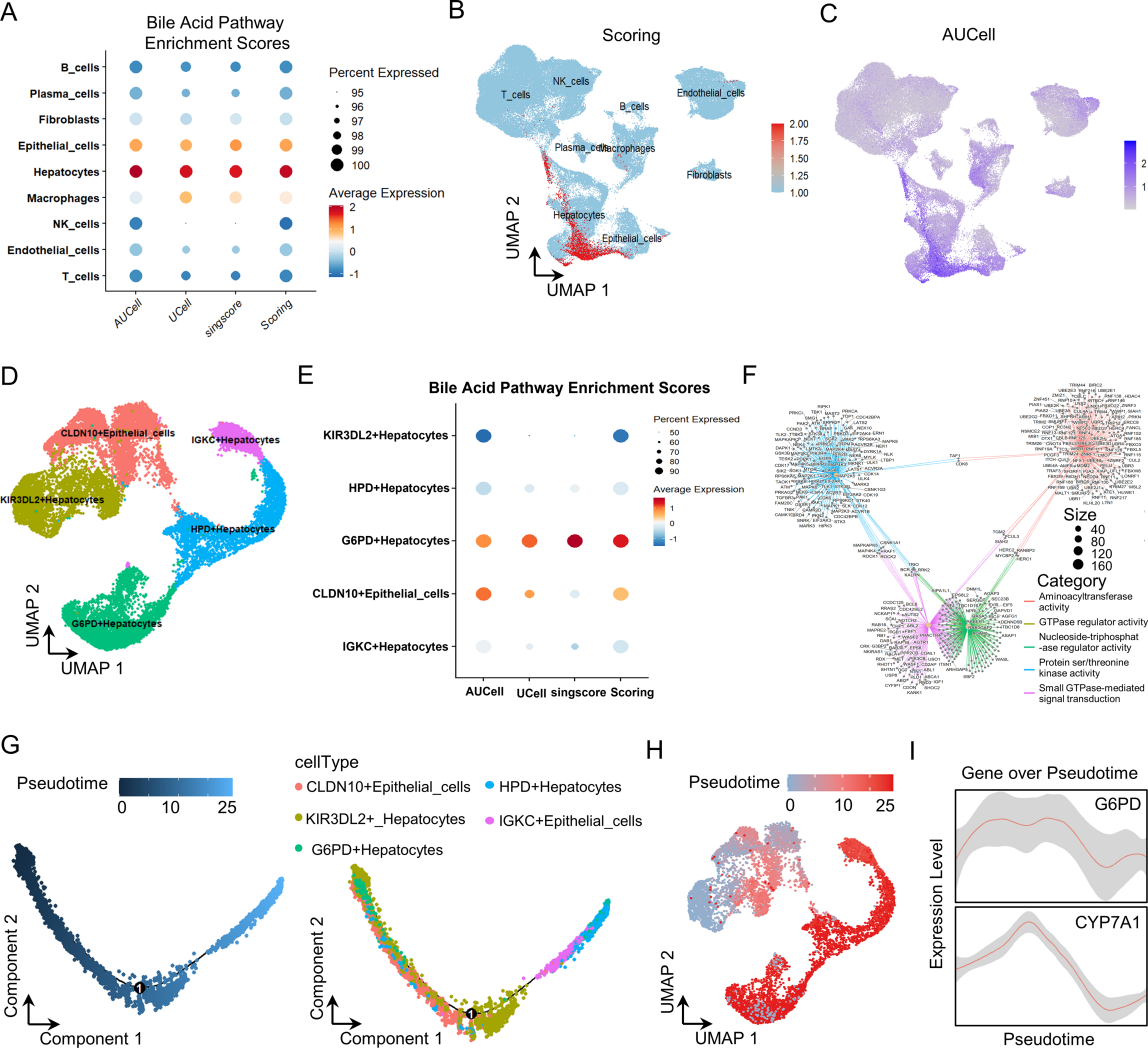
Bile acid metabolic features in scRNA-seq analysis of HCC patients. **(A)** Activity scores of bile acid metabolism across different cell types. **(B, C)** UMAP visualization of bile acid metabolism scores stratified by cell type. **(D)** UMAP projection depicting malignant and epithelial subclusters. **(E)** Comparative analysis of bile acid metabolism scores between malignant and epithelial subpopulations. **(F)** Network representation of enriched signaling cascades in G6PD^+^ malignant cells. **(G)** Pseudotime trajectory reconstruction of malignant cell subpopulations. **(H)** Mapping of differentiation states within the G6PD^+^ malignant cell cluster. **(I)** Dynamic expression patterns of G6PD^+^ malignant cell markers along the differentiation trajectory.

Our single-cell bile acid metabolism profiling revealed pronounced metabolic heterogeneity across cellular populations, with malignant cells exhibiting the highest bile acid metabolism scores (**Figures 2A–C**). Dimensionality reduction and clustering analyses further identified five distinct malignant cell subtypes, among which G6PD^+^ malignant cells displayed a uniquely elevated bile acid metabolic signature (**Figures 2D, E**; **Supplementary Figure S1**). Pathway enrichment analysis demonstrated significant activation of bile acid–related signaling pathways within this metabolically distinct subpopulation (**Figure 2F**). Pseudotime trajectory analysis positioned the G6PD^+^ malignant cells at a critical transitional state within the malignant cells differentiation continuum (**Figures 2G, H**), showing a dynamic expression pattern closely paralleling that of the characteristic bile acid–synthesizing enzyme CYP7A1 (**Figure 2I**).Collectively, these findings suggest that G6PD^+^ malignant cells represent a pivotal subpopulation in the regulation of bile acid metabolism, providing novel mechanistic insight into the metabolic dysregulation underlying HCC progression.

### 3.3 Spatial niches of bile acid metabolism

**Figure 3:**
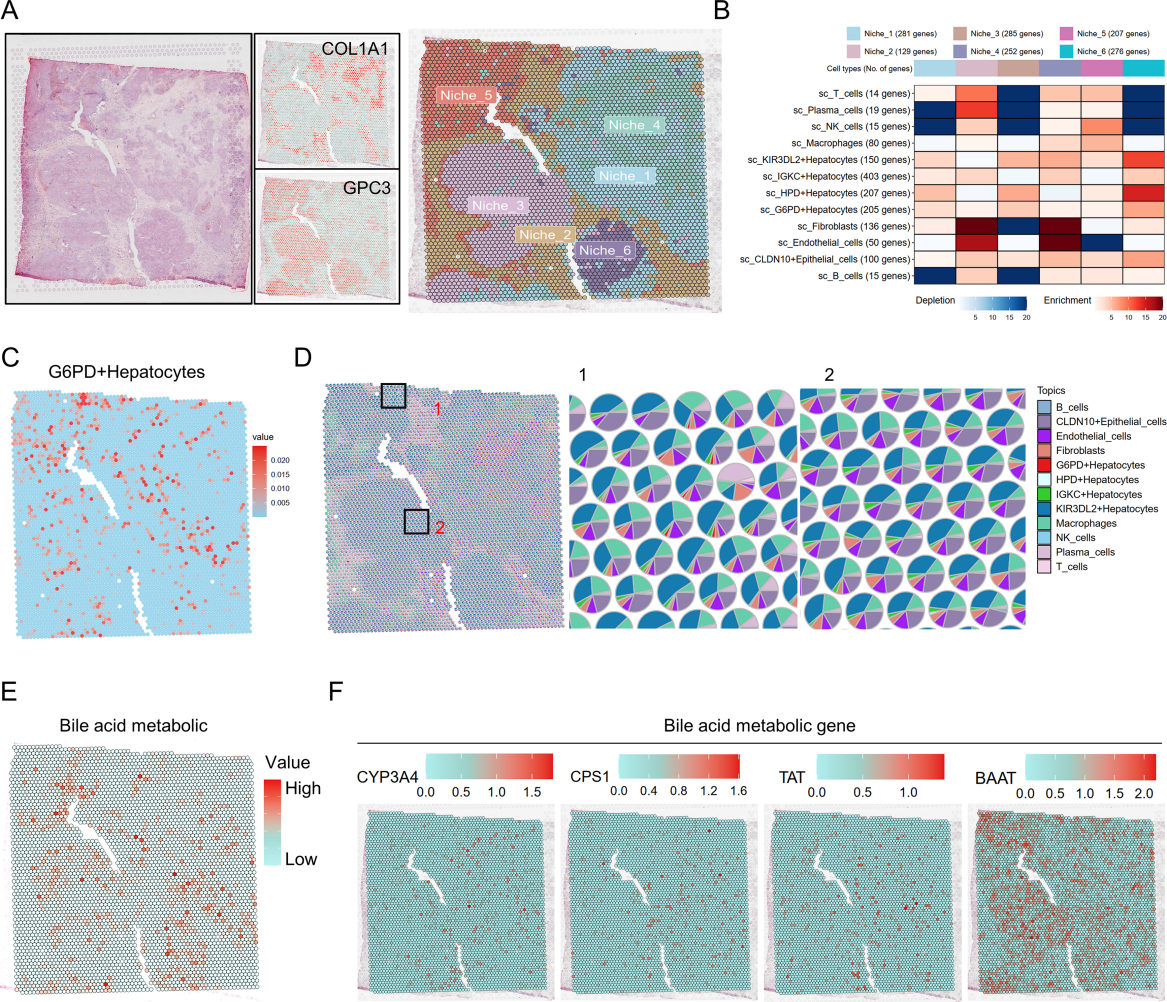
Spatial transcriptomic analysis of bile acid metabolic features in G6PD^+^ malignant cells. **(A)** Spatial imaging and regional annotation of HCC tissue sections. **(B)** Multimodal Intersection Analysis (MIA) of cell enrichment across different spatial regions. **(C)** Spatial distribution pattern of G6PD^+^ malignant cell populations within tumor tissues. **(D)** Cellular composition map of tumor microenvironmental domains enriched in G6PD^+^ malignant cells. **(E, F)** Spatial expression profiles of bile acid metabolism–related genes within tumor regions.

To characterize the spatial distribution patterns of bile acid metabolism, we performed spatial transcriptomic analysis using dataset 226334_D5_2. Spatial imaging and regional annotation identified seven distinct microenvironmental regions within HCC tissues (**Figure 3A**). Cell enrichment analysis based on the Multimodal Intersection Analysis (MIA) algorithm revealed that Region 4 represented a key area enriched with G6PD^+^ malignant cells (**Figure 3B**). Spatial pattern analysis demonstrated that G6PD^+^ malignant cells were non-uniformly distributed across the tumor tissue (**Figure 3C**), showing marked accumulation in Region 4, which displayed a complex composition of multiple cell types (**Figure 3D**). Notably, key genes involved in bile acid metabolism, including CYP7A1, CYP27A1, NR1H4, CYP7B1, and BSEP, exhibited significantly variable expression levels among these spatial domains (**Figures 3E, F**), indicating pronounced spatial heterogeneity of bile acid metabolism within the tumor microenvironment. Collectively, these findings suggest that bile acid metabolism in G6PD^+^ malignant cells may play a region-specific role within distinct spatial niches of the HCC microenvironment.

### 3.4 Construction of a risk prediction model using machine learning

**Figure 4:**
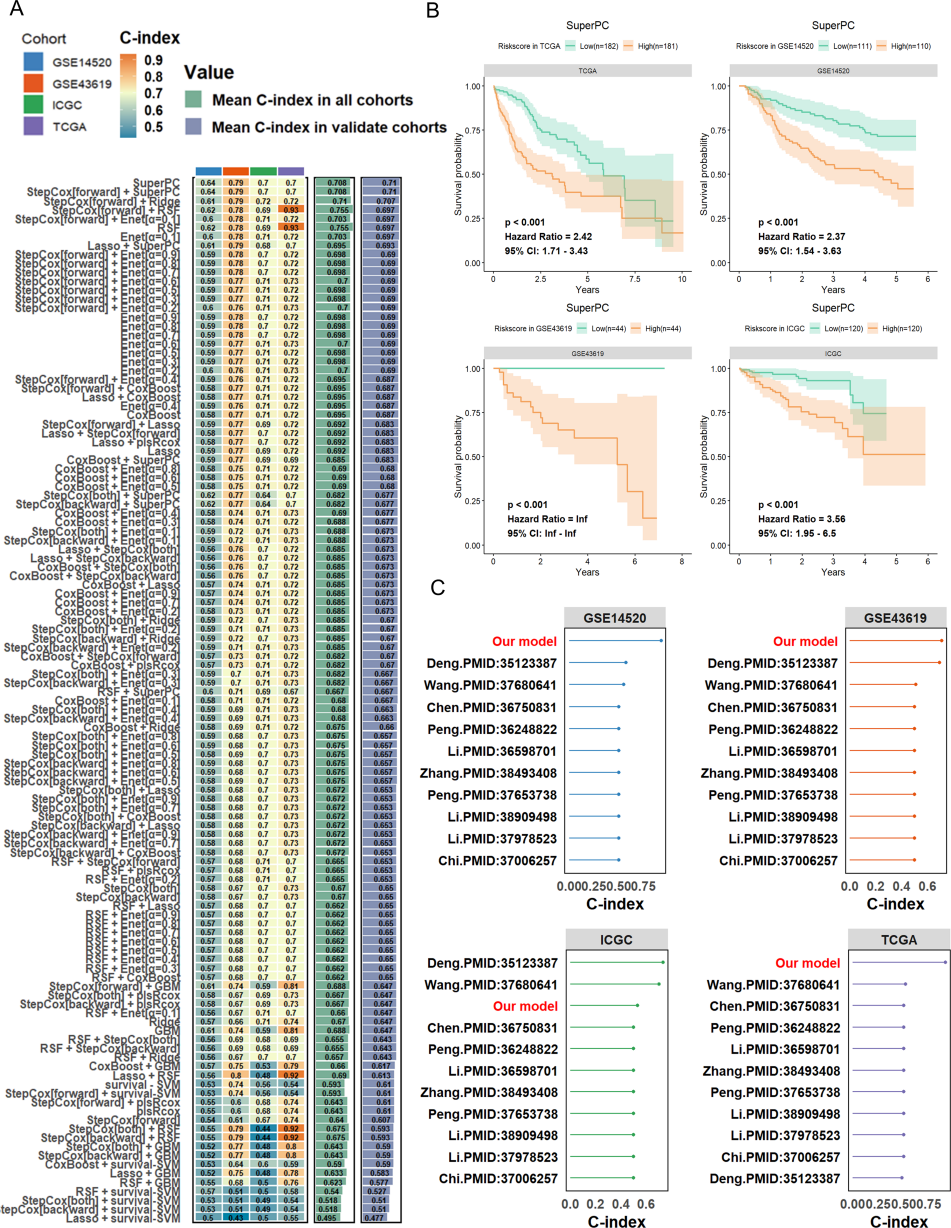
Construction of a bile acid metabolism–based risk scoring model derived from G6PD^+^ malignant cells using integrated machine learning. **(A)** Risk assessment of different models across four independent cohorts evaluated by concordance index (C-index). **(B)** Comparative survival analysis between high-risk and low-risk patient subgroups. **(C)** Performance comparison between the G6PD^+^ malignant cell–based predictive model and previously established published prognostic models.

To develop a robust prognostic model, we integrated bile acid metabolism–related features using multiple machine learning algorithms. The results demonstrated that different algorithmic combinations yielded varying concordance index (C-index) values across cohorts, with our integrated model achieving superior predictive performance in all datasets (**Figure 4A**). Survival analyses across four independent cohorts—TCGA, GSE14520, GSE43619, and ICGC—consistently revealed that patients in the high-risk group had significantly worse overall survival compared to those in the low-risk group (*p* < 0.001), with corresponding hazard ratios (HRs) of 2.42, 2.37, infinity, and 3.56, respectively (**Figure 4B**). When compared with existing prognostic models, our model achieved consistently higher C-index values across multiple datasets, underscoring its superior prognostic accuracy (**Figure 4C**). These findings indicate that the bile acid metabolism–based risk scoring model can effectively predict clinical outcomes in HCC patients, thereby providing valuable guidance for precision prognosis assessment and clinical decision-making.

### 3.5 Performance evaluation of the G6PD^+^ malignant cell–based predictive model

**Figure 5:**
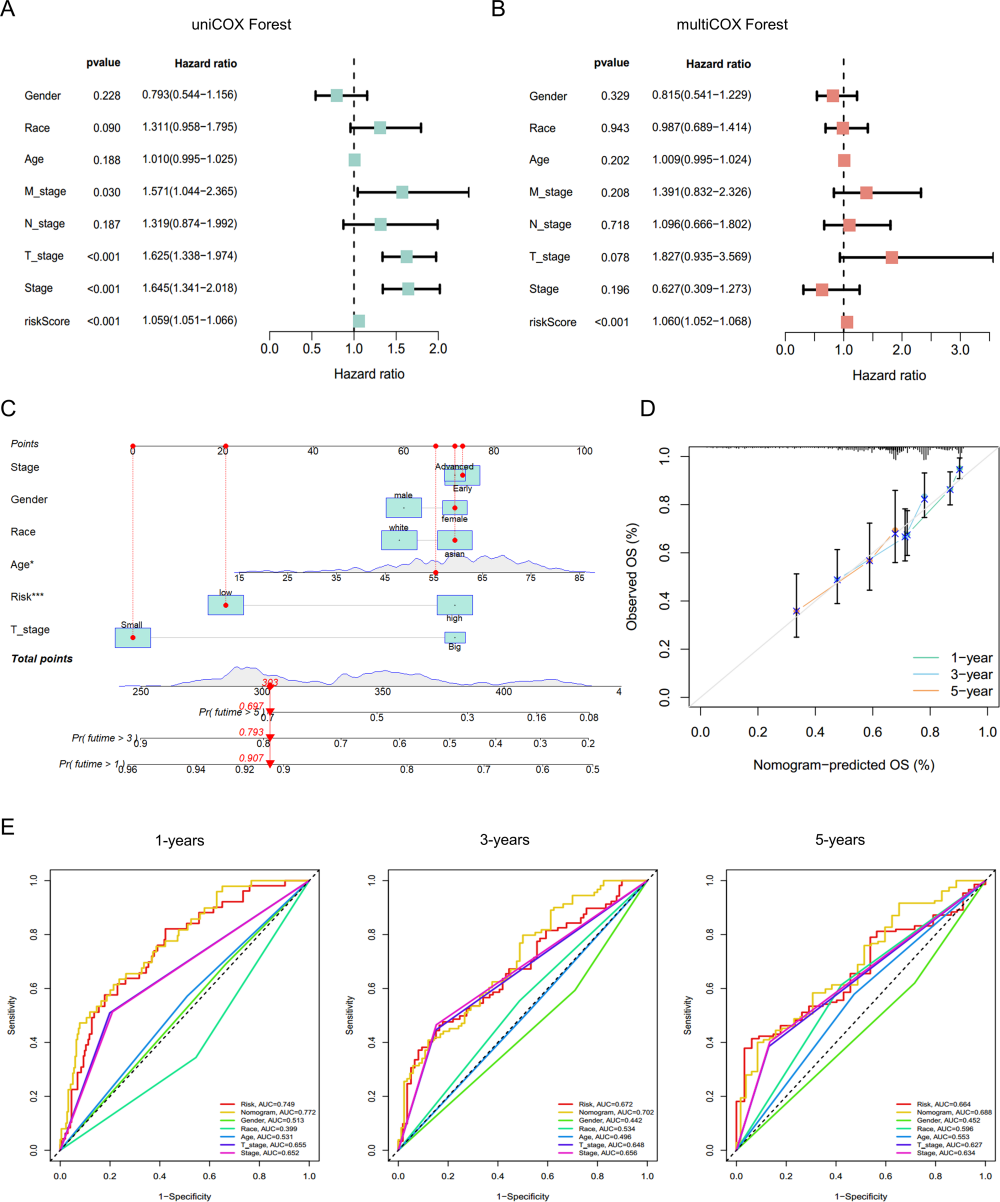
Validation of the independent prognostic value of the bile acid metabolism–based risk score. **(A)** Univariate Cox regression analysis of clinicopathological parameters and the G6PD^+^ malignant cell–derived risk score. **(B)** Multivariate Cox regression analysis assessing the independence of the G6PD^+^ malignant cell–based risk score from clinical covariates. **(C)** Nomogram integrating common clinical parameters with the G6PD^+^ malignant cell–based risk score for prognostic prediction. **(D)** Calibration curve evaluating the predictive accuracy of the nomogram. **(E)** Time-dependent ROC curves illustrating the predictive performance of the model at 1-, 3-, and 5-year survival intervals.

To validate the independent prognostic value of the bile acid metabolism–derived risk score, we performed univariate and multivariate Cox regression analyses. Univariate analysis revealed that, apart from sex, race, age, and N stage, variables such as M stage, T stage, overall clinical stage, and the risk score were all significant prognostic factors (**Figure 5A**). Multivariate analysis further confirmed that the risk score remained an independent prognostic indicator after adjustment for other clinical covariates (*p* < 0.001, HR = 1.060, 95% CI: 1.052–1.068) (**Figure 5B**). Based on these findings, we constructed an integrated nomogram combining the risk score with key clinical parameters (**Figure 5C**), which demonstrated excellent calibration performance (**Figure 5D**). Time-dependent ROC curve analysis showed that our model outperformed other clinical variables in predicting 1-, 3-, and 5-year survival, achieving AUC values of 0.730, 0.752, and 0.689, respectively (**Figure 5E**). Collectively, these results establish the bile acid metabolism–based risk score as an independent and powerful tool for prognostic assessment in HCC.

### 3.6 Distinct molecular characteristics between risk groups

**Figure 6:**
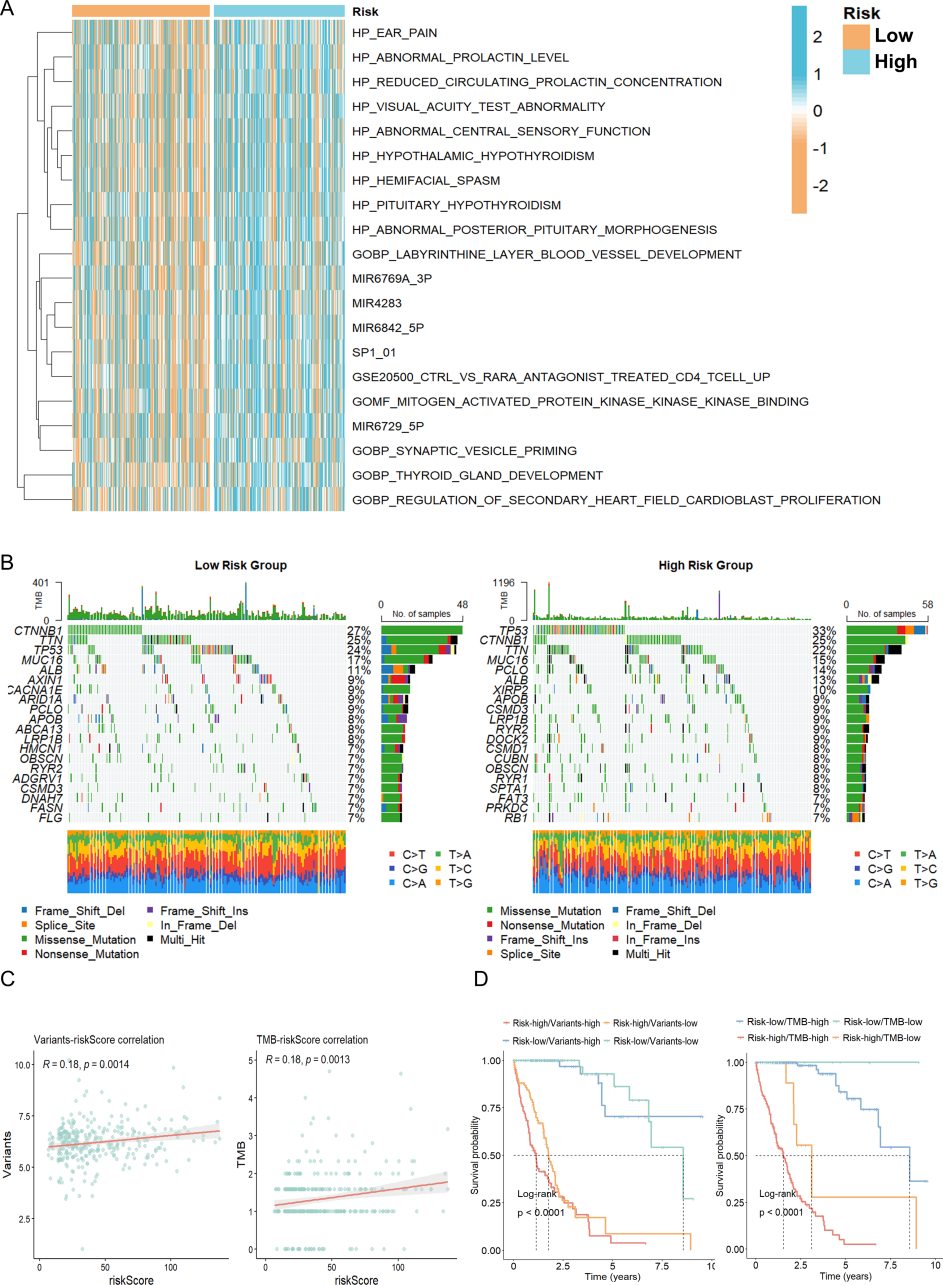
Molecular characteristics of risk groups based on G6PD^+^ malignant cells. **(A)** Comparative pathway enrichment analysis between high-risk and low-risk patient groups. **(B)** Mutation landscape analysis illustrating genomic alterations across high- and low-risk groups. **(C)** Correlation analysis between the G6PD^+^ malignant cell–derived risk score and tumor mutation burden (TMB). **(D)** Survival analysis stratified by combined risk score and TMB status.

Comparative analyses between risk groups revealed substantial differences in molecular and clinical features. Pathway enrichment analysis indicated that pro-tumorigenic signaling pathways were significantly activated in the high-risk group (**Figure 6A**). Mutation profiling demonstrated a higher TP53 mutation frequency among high-risk patients (**Figure 6B**), accompanied by a markedly elevated tumor mutation burden (TMB) (*p* < 0.001; **Figure 6C**). Both high-risk classification and increased TMB were associated with significantly poorer overall survival (**Figure 6D**), thereby validating the prognostic reliability of our risk stratification framework. Collectively, these findings provide a molecular and pathological basis for bile acid metabolism–related risk stratification in HCC, highlighting its potential utility in guiding prognosis and individualized therapeutic strategies.

### 3.7 Immune infiltration characteristics of risk subgroups

**Figure 7:**
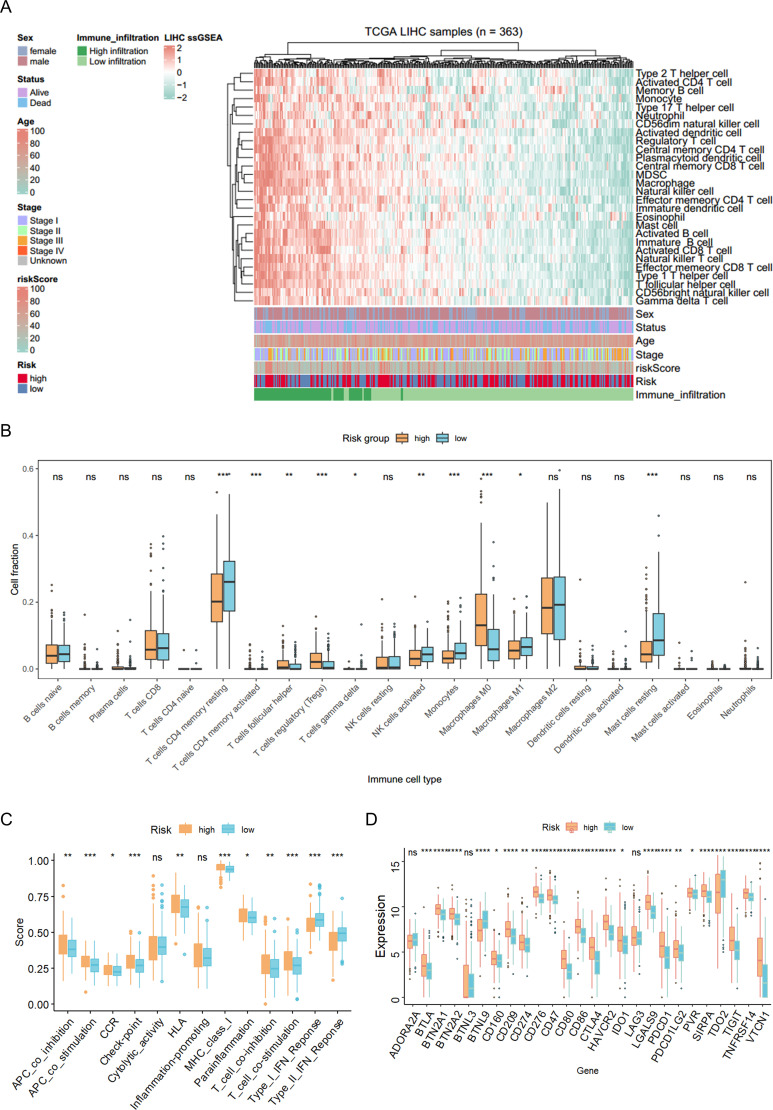
Immune infiltration characteristics of risk groups based on G6PD^+^ malignant cells. **(A)** Immune infiltration landscape of HCC patients. **(B)** Comparative analysis of immune cell infiltration between high-risk and low-risk groups. **(C)** Functional characterization of immune responses in high- and low-risk cohorts. **(D)** Differential expression patterns of immune checkpoint molecules across risk groups. *p < 0.05, **p < 0.01, ***p < 0.001, ns > 0.05.

To characterize the immune microenvironment associated with G6PD^+^ malignant cells, we conducted a comprehensive immune analysis using single-sample gene set enrichment analysis (ssGSEA). Distinct immune landscapes were observed between the risk subgroups (**Figure 7A**), with the high-risk group showing significant enrichment of specific immune cell populations, including various T-cell subsets, macrophage subtypes, and other immune cells (**Figure 7B**). Pathway enrichment analysis further revealed pronounced differences in immune-related functional pathways among high-risk patients (**Figure 7C**), accompanied by differential expression of immune checkpoint molecules (**Figure 7D**). Collectively, these findings suggest that G6PD^+^ malignant cells with aberrant bile acid metabolism may contribute to HCC progression by modulating the immune microenvironment, offering mechanistic insight into the interplay between metabolic reprogramming and immune regulation in HCC.

### 3.8 Strong interactions between G6PD^+^ malignant cells and endothelial cells in aberrant bile acid metabolism

**Figure 8:**
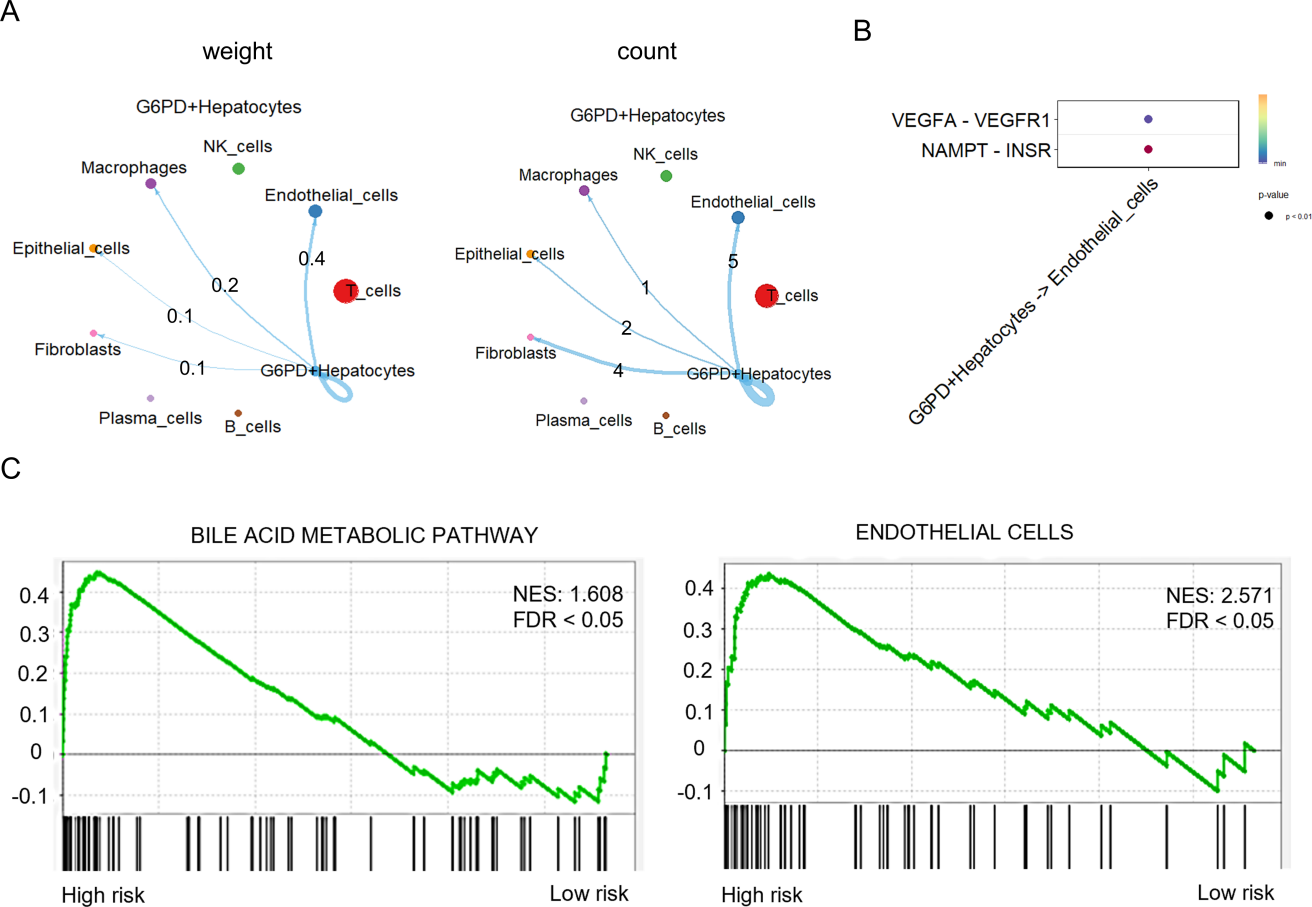
Roles of bile acid metabolism and specific immune cell pathways in HCC prognosis. **(A)** Cell–cell communication network between G6PD^+^ malignant cells and other cell populations within the HCC microenvironment. **(B)** Signaling crosstalk between G6PD^+^ malignant cells and endothelial cells, highlighting activation of the VEGFA–VEGFR1 and NAMPT–INSR pathways. **(C)** GSEA analysis demonstrating the correlation between the G6PD^+^ malignant cell–derived risk score and pathways related to bile acid metabolism and endothelial cell function.

CellChat analysis revealed that G6PD^+^ malignant cells exhibited the strongest intercellular interactions with endothelial cells (**Figure 8A**), accompanied by significant activation of the VEGFA–VEGFR1 and NAMPT–INSR signaling pathways (**Figure 8B**). Consistently, GSEA analysis demonstrated that the high-risk signature was closely associated with specific signaling pathways involving bile acid metabolism and endothelial cell regulation (**Figure 8C**). Both pathways were significantly enriched in the high-risk group, suggesting a potential synergistic role in promoting HCC progression. These findings confirm that G6PD^+^ malignant cells with aberrant bile acid metabolism may drive HCC progression by reshaping tumor microenvironmental signaling networks, particularly through the enhancement of angiogenic activity.

### 3.9 BIBR-1532 as a potential therapeutic compound targeting G6PD^+^ malignant cells

**Figure 9:**
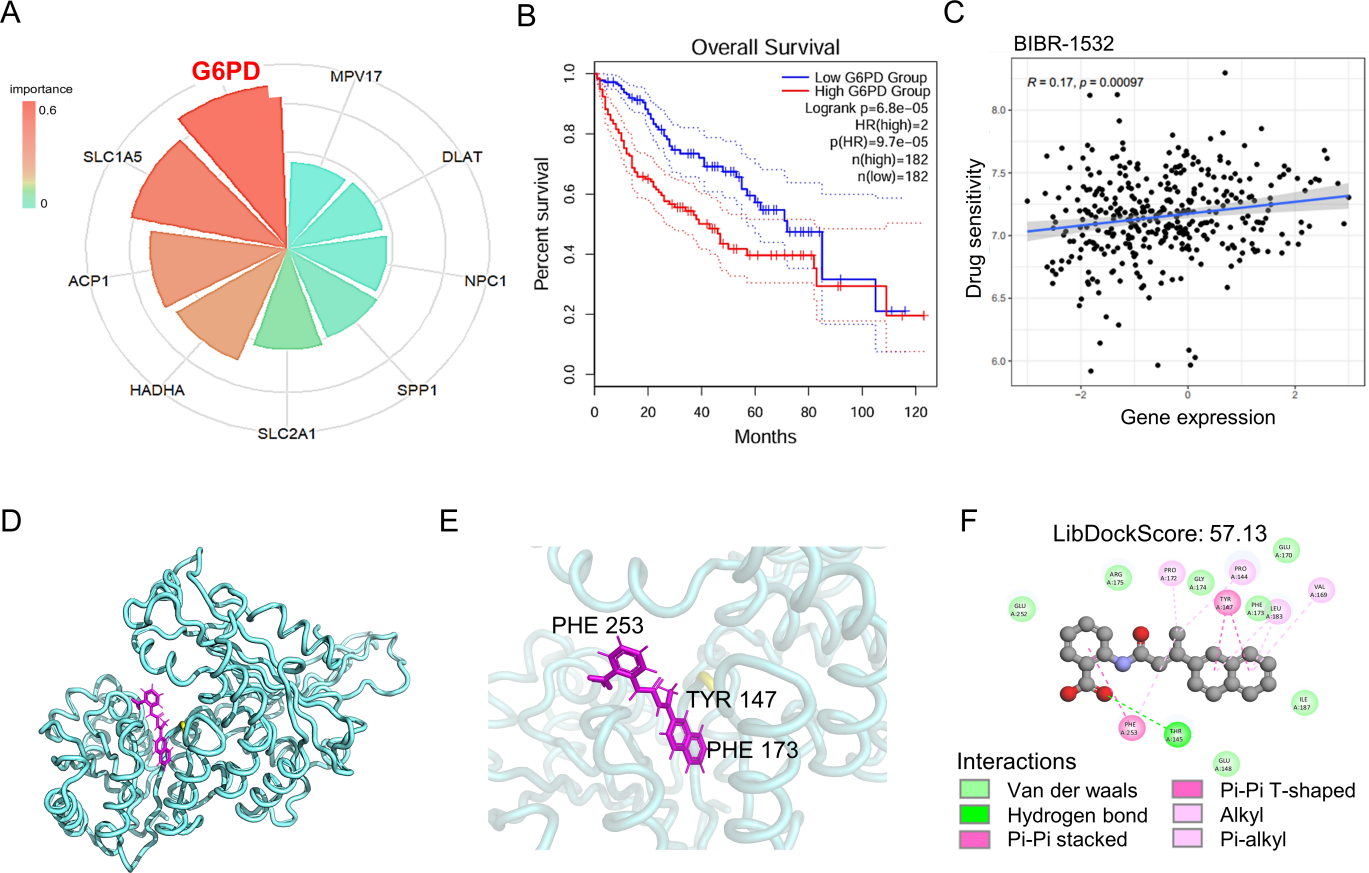
G6PD as a key regulator and potential drug target in bile acid metabolism. **(A)** Feature importance analysis of genes in the risk prediction model. **(B)** Survival outcomes stratified by G6PD expression levels. **(C)** Correlation analysis between G6PD expression and therapeutic drug sensitivity. **(D)** Molecular docking simulation of BIBR-1532 with the G6PD-encoded protein. **(E)** Structural characterization of the BIBR-1532–G6PD binding interactions. **(F)** Two-dimensional visualization of the binding interface between BIBR-1532 and G6PD.

Based on machine learning–driven gene importance analysis, G6PD was identified as the most critical regulatory factor (**Figure 9A**), with its elevated expression strongly correlated with poor prognosis in HCC patients (**Figure 9B**). Drug sensitivity analysis further revealed a significant association between G6PD expression and the anticancer response to BIBR-1532 (**Figure 9C**). Molecular docking simulations confirmed that BIBR-1532 forms stable interactions with the G6PD protein through key residues PHE173, TYR147, and PHE253 (**Figures 9D–F**), suggesting a high binding affinity and potential inhibitory effect. Collectively, these findings provide a theoretical foundation for precision therapeutics targeting G6PD, highlighting BIBR-1532 as a promising candidate for the treatment of HCC driven by aberrant bile acid metabolism.

### 3.10 *In Vitro* validation and functional characterization of G6PD

**Figure 10:**
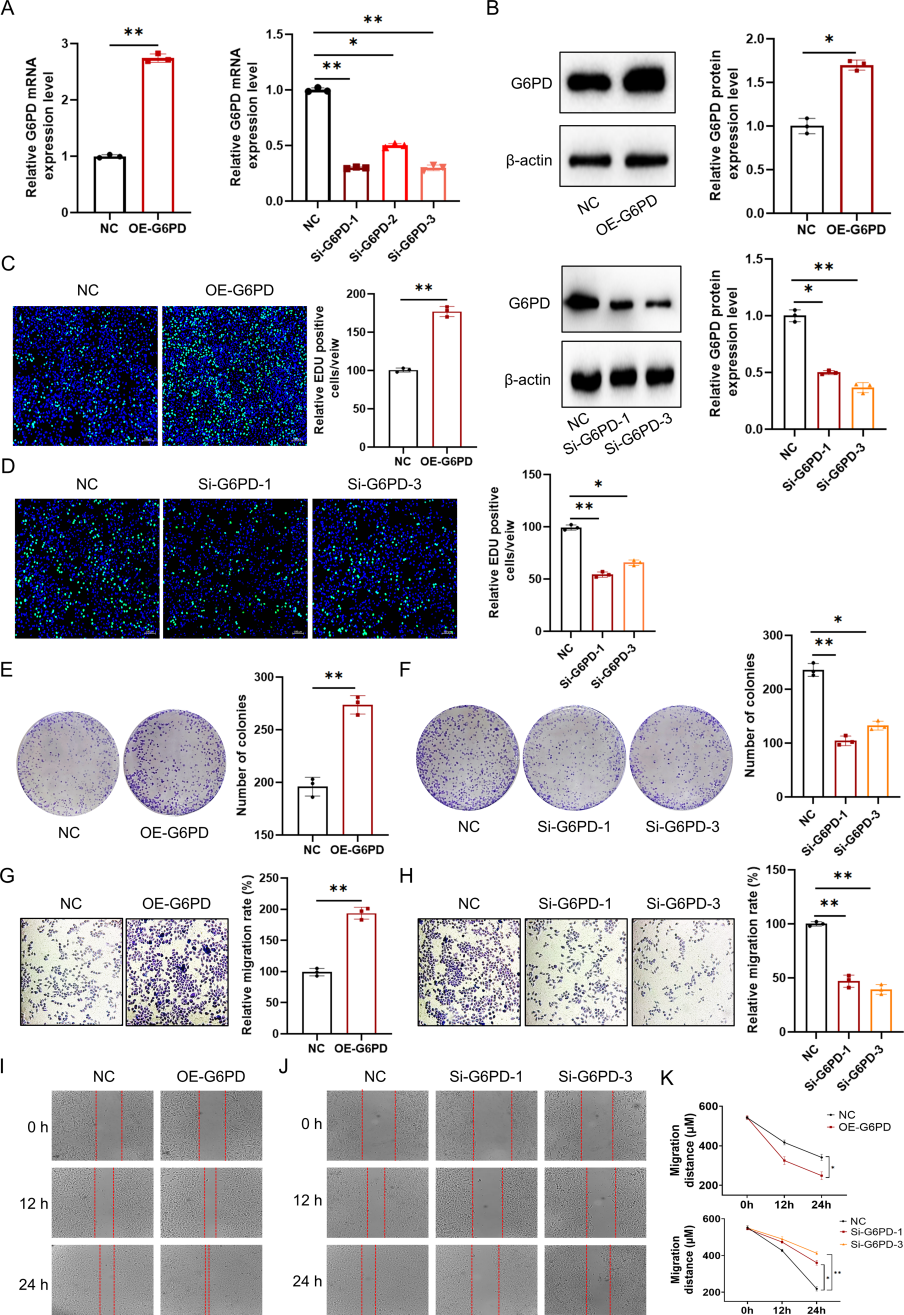
Functional validation of G6PD in HCC cells. **(A)** qRT-PCR analysis confirming the efficiency of G6PD overexpression and knockdown in HCCLM3 cells (n = 3). **(B)** Western blot validation of G6PD protein expression levels following overexpression or silencing in HCCLM3 cells (n = 3). **(C, D)** EdU assays evaluating the proliferative capacity of HCCLM3 cells after G6PD interference (n = 3). **(E, F)** Colony formation assays assessing the tumorigenic potential of HCCLM3 cells after G6PD knockdown (n = 3). **(G, H)** Transwell invasion assays evaluating the invasive capacity of HCCLM3 cells after G6PD knockdown or overexpression (n = 3). **(I–K)** Wound-healing assays examining the migratory ability of HCCLM3 cells following G6PD modulation (n = 3). *p < 0.05, **p < 0.01.

To verify the functional role of G6PD in HCC progression, we performed a series of *in vitro* experiments. First, qRT-PCR confirmed the efficiency of both G6PD overexpression and knockdown (**Figure 10A**). Western blot analysis further validated corresponding changes in protein expression—G6PD protein levels were markedly increased in the OE-G6PD group, whereas significantly reduced expression was observed in the Si-G6PD-1 and Si-G6PD-3 groups (**Figure 10B**). Cell proliferation assays demonstrated that G6PD overexpression promoted proliferation, reflected by stronger fluorescence intensity signals (**Figure 10C**), while G6PD knockdown significantly inhibited HCC cell growth (**Figure 10D**). Colony formation assays further confirmed that G6PD knockdown (Si-G6PD-1 and Si-G6PD-3) markedly reduced colony numbers, whereas overexpression of G6PD enhanced colony-forming ability (**Figures 10E, F**). Transwell invasion assays revealed that G6PD knockdown (Si-G6PD-1 and Si-G6PD-3) led to a substantial reduction in the number of invading cells, while the OE-G6PD group exhibited a marked increase in invasion potential (**Figures 10G, H**). Similarly, silencing G6PD notably impaired cell migratory capacity compared with controls, whereas overexpression significantly enhanced migration (**Figures 10I–K**). Collectively, these findings demonstrate that G6PD^+^ malignant cells with aberrant bile acid metabolism promote HCC progression by enhancing tumor cell proliferation, migration, and invasion capabilities, thereby underscoring G6PD as a functional driver and potential therapeutic target in HCC.

## 4 Discussion

Tumor tissues exhibit pronounced spatial heterogeneity, most notably at the invasive front where cells interacting with the extracellular matrix and vasculature display enhanced invasiveness and metastatic capacity (26, 27). In our analysis, we identified a population of G6PD^+^ malignant cells with aberrant bile acid metabolism that are enriched at the tumor boundary regions of HCC. These cells exhibit high G6PD expression, indicating a hyperactivated pentose phosphate pathway (PPP) that provides sufficient NADPH to counter oxidative stress and support biosynthetic demands. This metabolic feature likely reflects adaptation to a bile acid–rich, inflammatory microenvironment, where excessive bile acids induce ROS accumulation, compelling tumor cells to upregulate G6PD and antioxidant defenses (28, 29). Notably, the enrichment of G6PD^+^ cells at the invasive front suggests their potential role as drivers of invasion and dissemination (30). Previous studies have shown that elevated G6PD expression drives malignant phenotypes in HCC, including enhanced migration, invasion, and EMT (31). Our findings are consistent with these reports, implying that G6PD^+^ tumor cells at the periphery may act as the “vanguard” of tumor expansion.

Dysregulated bile acid metabolism not only induces hepatotoxic accumulation and inflammation but also affects tumor cell behavior via multiple signaling pathways. In HCC, elevated bile acids can activate inflammation-related cascades such as NF-κB and JAK–STAT3, as well as metabolic sensing pathways including Hippo–YAP and AMPK (32–34),indicating extensive crosstalk between bile acid signaling and proliferative signaling. Meanwhile, bile acid receptors such as TGR5 and FXR regulate both lipid and glucose metabolism, linking metabolic and oncogenic signaling (35, 36). Excessive bile acid activates GPBAR1 specifically in cancer-associated fibroblasts, prompting CXCL10 production that enhances EMT and metastasis in cholangiocarcinoma (37). This process also recruits neutrophils to establish an immunosuppressive microenvironment. Importantly, inhibiting bile acid synthesis in liver cancer cells reprograms this milieu, reinvigorating T-cell responses, suppressing tumor growth, and consequently sensitizing tumors to anti-PD-1 immunotherapy (38). While previous studies have confirmed the role of disordered bile acid metabolism in HCC progression at the molecular level, our research focuses on the G6PD^+^ malignant cellular subtype with aberrant bile acid metabolism and its functional impact, thereby further substantiating the critical role of bile acid metabolism in HCC progression at the cellular level.

Importantly, glucose-6-phosphate dehydrogenase (G6PD), the rate-limiting enzyme of the PPP, may itself be regulated by bile acid–related oxidative signaling. Oxidized bile acids can induce ROS production and activate transcription factors like NRF2, which transcriptionally upregulates G6PD to enhance redox balance (28, 29). Mice deficient in NRF2 exhibit reduced HCC incidence in carcinogenic models, partially due to decreased G6PD expression and PPP activity, leading to uncompensated oxidative stress (28). These findings imply that bile acid–induced oxidative stress promotes tumor survival through the NRF2–G6PD axis, forming a feedback loop that links bile acid and glucose metabolism. We propose that metabolic crosstalk drives HCC progression by enhancing tumor cell invasiveness and anti-apoptotic resistance, allowing them to thrive in harsh microenvironments.

HCC is a hypervascular tumor, whose growth and metastasis are tightly dependent on vascular remodeling, including both classical angiogenesis and non-classical vasculogenic mimicry (VM) formation (39–41). We observed that G6PD^+^ malignant cells with aberrant bile acid metabolism maintain intimate interactions with endothelial cells and macrophages in the tumor microenvironment. At the invasive margin, G6PD^+^ malignant cells are often spatially adjacent to endothelial cells, suggesting direct cellular crosstalk. On one hand, these tumor cells may secrete pro-angiogenic factors such as VEGF and basic fibroblast growth factor (bFGF) to stimulate endothelial proliferation and neovascularization (42, 43). Conversely, endothelial cells release pro-invasive cues, enhancing tumor cell motility and invasiveness (42, 44, 45). This bidirectional feedback facilitates mutual activation between invasive-front tumor cells and neighboring endothelium, thereby driving angiogenesis and local invasion.

Macrophages likewise play pivotal roles in angiogenesis and immune remodeling of the tumor microenvironment (46, 47). We speculate that metabolic byproducts derived from G6PD^+^ malignant cells may drive macrophage M2 polarization, promoting secretion of pro-angiogenic and immunosuppressive mediators. Previous studies have shown that under conditions of bile acid dysregulation, tumor-derived inflammatory mediators (e.g., SAA proteins) recruit macrophages and bias them toward an M2 phenotype (30). This suggests that bile acid imbalance modulates the malignant cell–macrophage axis, reshaping both immune and vascular niches. In our results, the interaction between G6PD^+^ malignant cells and macrophages was particularly strong, implying that G6PD^+^ cells may secrete cytokines that attract macrophages to the tumor margin. The recruited macrophages, in turn, release growth factors and cytokines that further enhance angiogenesis and invasion (48). Collectively, G6PD^+^ malignant cells with aberrant bile acid metabolism interact dynamically with endothelial and immune cells, establishing a pro-angiogenic and immunosuppressive microenvironment that supports HCC progression. Therefore, targeting these intercellular interactions or blocking key pro-angiogenic signals may represent promising strategies to impede vascular remodeling and metastatic potential in HCC.

Our study identifies a promising bile acid metabolism-related prognostic signature, yet its clinical application faces two main challenges. First, standardization is difficult due to technical variations in RNA-seq and sample processing, as well as biological heterogeneity in ethnicity, lifestyle, and gut microbiota, which may affect signature performance. Second, the model requires broader validation, and multi-center evaluation. Moreover, since bile acid metabolism influences multiple cancers, testing this signature across tumor types will clarify whether it serves as a pan-cancer biomarker or a context-specific tool, guiding its use in precision oncology.

Nevertheless, several limitations should be acknowledged. First, the causal link between G6PD^+^ malignant cells and bile acid metabolic dysregulation remains correlative; functional studies are needed to confirm whether bile acid signaling directly modulates G6PD activity and its oncogenic consequences. Second, while spatial analyses revealed G6PD^+^ cell enrichment at the tumor margin, it remains unclear whether this is a universal phenomenon across different HCC stages and differentiation states—requiring validation in larger cohorts. Third, therapeutic targeting of metabolism must consider potential safety constraints; for example, G6PD inhibition may trigger hemolysis, particularly in individuals with underlying G6PD deficiency. Thus, achieving tumor-specific targeting while sparing normal tissues remains a key challenge. Moreover, all functional assays for proliferation, migration, and invasion were conducted using a single HCC cell line (HCCLM3). While this line was selected for its aggressive metastatic phenotype, which aligns with our research focus on progression, conclusions drawn from a single model system require caution in generalization to the highly heterogeneous landscape of HCC. Finally, the bile acid–immune–metabolic interplay is exceedingly complex; its dual (“double-edged sword”) effects warrant balanced consideration. Future studies should leverage multi-omics and spatial single-cell technologies to map the bile acid–metabolism–immunity network, identify critical nodes, and guide the discovery of precise intervention targets and specific regulatory mechanism. Future validation may involve a multicenter retrospective cohort with broader clinical features, combined with PDOX models derived from co-cultured patient derived organoids and autologous immune cells.

In summary, this study definitively identifies a G6PD^+^ malignant hepatocyte subset with reprogrammed bile acid metabolism, situated within immunosuppressive and angiogenic niches, and establishes its derived gene signature as a robust, independent prognostic indicator for HCC. Looking forward, integrating metabolic-targeted approaches with current treatment paradigms, guided by cross-disciplinary strategies, may open new avenues for precision therapy in HCC and improve patient survival outcomes.

## Data Availability

The data analyzed in this study are publicly available and were obtained from public repositories. Processed data and analysis results generated in this study are provided in the article and Supplementary Material. Further inquiries can be directed to the corresponding authors.
