# Supplementary material for: Single-cell RNA sequencing and spatial transcriptomic analysis reveal a distinct population of G6PD+ cells with aberrant bile acid metabolism in hepatocellular carcinoma
Source: Front Immunol. 2026 Feb 2;17:1739293. doi: 10.3389/fimmu.2026.1739293 (PMC12907316; doi:10.3389/fimmu.2026.1739293)
Supplement: Supplementary file 1 [file Table1.docx]

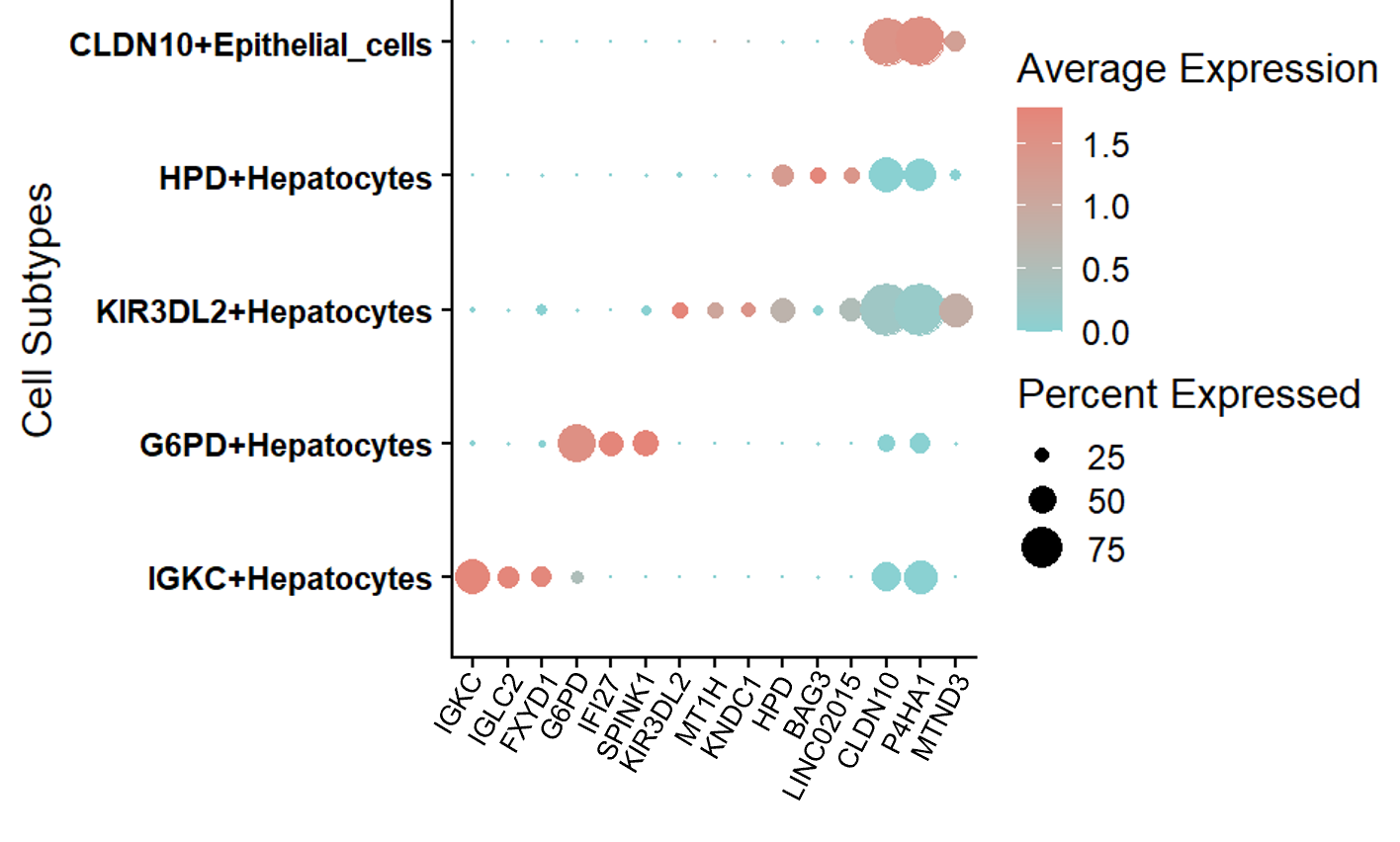


Supplementary Fig. S1. Bubble plot illustrating the expression of marker genes across identified hepatocyte cell subtypes.
